# Malignant Pheochromocytoma Presenting as Acute Coronary Syndrome: A Report of a Rare Case

**DOI:** 10.7759/cureus.83473

**Published:** 2025-05-04

**Authors:** Saif M Srouji, Nicholas Reyes, Rukhsar Nadeem, Nathanael A Reyes, Sukhampal S Sidhu, Mehdi Hakimipour

**Affiliations:** 1 Internal Medicine, Saint Agnes Medical Center, Fresno, USA; 2 Endocrinology, Saint Agnes Medical Center, Fresno, USA

**Keywords:** adrenal disease, adrenal pheochromocytoma, cardio vascular disease, coronary artery disease, ecg abnormalities, electrocardiography (ecg), outcomes of hypertensive emergency, pheochromocytoma crisis, st-elevation myocardial infarction (stemi)

## Abstract

Pheochromocytomas are rare tumors of the adrenal medulla. The classic presentation for this condition consists of a triad of symptoms: headache, tachycardia, and sweating in an episodic fashion. Rarely, pheochromocytomas are associated with cardiac pathology specifically acute coronary syndrome (ACS) and cardiomyopathy, which is usually attributed to catecholamine excess. They might also present secondary to elevations in blood pressure or arrhythmias. ECG changes are most often encountered as sinus tachycardia. We present a case of previously undiagnosed pheochromocytoma that showed up as ACS across multiple admissions in the setting of significant obstructive coronary artery disease. We aim to contribute to the growing literature reporting this uncommon presentation in order to showcase these cases to the medical audience.

## Introduction

Pheochromocytomas are rare tumors of the adrenal medulla that arise from chromaffin cells and secrete catecholamines. It is estimated that the annual incidence of pheochromocytomas is approximately 0.8 per 100,000 person-years [1]. The classic triad of symptoms is headache, sweating, and tachycardia that occur in an episodic fashion; however, only about 25% of patients experience these symptoms in unison [2,3]. Other clinical features include palpitations, chest pain, and anxiety. Pheochromocytomas have been recognized to cause various cardiovascular complications including tachycardia, tachyarrhythmias, cardiomyopathies, hypertensive crisis, and acute coronary syndrome (ACS) [4]. These symptoms are believed to be caused by excessive secretion of metanephrines and catecholamines [5]. After conducting a literature review, it remains unclear how many ACS cases occur as a direct result of pheochromocytoma catecholamine surge, as opposed to those occurring due to existing asymptomatic coronary artery disease (CAD) that happens to be diagnosed after angiographic testing during a catecholamine surge episode. Initial biochemical evaluation should include plasma-fractionated metanephrines or urinary-fractionated metanephrines. Subsequently, imaging including computed tomography (CT) of the abdomen and pelvis to locate the tumor is needed. Surgical resection is the definitive treatment of pheochromocytoma with alpha and beta blockade at least 7-14 days prior to surgical resection [6].

We report a case of pheochromocytoma that presented with recurrent paroxysmal symptoms of chest pain and palpitations associated with ST segment elevations on electrocardiography (ECG) in the setting of previously undiagnosed CAD. After multiple symptomatic presentations to the hospital, both a CT scan of the abdomen and an MRI were obtained that showed the presence of an adrenal mass. The patient was started on an alpha-blocker for symptomatic control leading up to surgical resection, after which surgical pathology confirmed the diagnosis. We anticipate that the associated catecholamine surge caused myocardial stress and led to myocardial damage evidenced by findings of ST elevation myocardial infarction (STEMI) on ECG. We also believe that this finding of pheochromocytoma resulted in earlier diagnosis of CAD that might have stayed silent until later stages had these episodes not occurred in association. The aim of this report is to describe how symptomatic pheochromocytoma resulted in earlier diagnosis of significant obstructive CAD, and how managing such cases could prove to be a challenge in and of itself, as there could be an overlap in symptomatology, possibly resulting in uncertainty and delay in the selection of a management approach. Moreover, invasive angiographic testing could prove to be challenging in the acute setting where adequate blood pressure control might be difficult, in addition to exposure to potential risks associated with such invasive procedures especially where the presence of preexisting asymptomatic CAD is not suspected. This could also impact the timing of definite surgical intervention.

## Case presentation

A 68-year-old Caucasian woman with a past medical history of primary hypertension, mixed hyperlipidemia, moderate obstructive sleep apnea, and non-insulin-dependent type 2 diabetes mellitus, initially presented to the emergency department (ED) with complaints of shortness of breath, presyncope, lightheadedness, and abdominal pain, stating these symptoms had been ongoing for several months prior to this presentation. She had been following up in the outpatient setting with her cardiologist; however, they were not able to attribute these symptoms to a cardiac-related cause based on outpatient evaluation. On presentation, her vital signs were notable for a heart rate (HR) of 112 beats/min, otherwise unremarkable. The initial laboratory analysis was remarkable as shown in Table 1.

**Table 1:** Initial laboratory investigation Initial laboratory investigation values were generally non-specific and were not diagnostic for the presentation.

Parameters	Patient Values	Reference Range
White blood cell count	11.3 K/mcL	4.5-11 K/mcL
Hemoglobin	11.1 g/dL	12-16 g/dL
Mean corpuscular volume	82 FL	81-99 FL
Platelet count	484 K/mcL	150-400 K/mcL
Blood urea nitrogen	25 mg/dL	8-21 mg/dL
Creatinine	1.04 mg/dL	0.5-1.2 mg/dL
Troponin	9 ng/L	0-14 ng/L
Brain natriuretic peptide	45 pg/mL	0-100 pg/mL

Physical exam in the ED was only significant for a reducible ventral hernia and an anxious-appearing individual. Chest x-ray (CXR) was negative for any acute cardiopulmonary process. Electrocardiogram (ECG) noted sinus tachycardia of 105 beats/min, no axis deviation, and no concerning ischemic ST segment changes. The patient was discharged home and recommended to follow up with her primary care physician (PCP) and cardiologist for further workup.

**Figure 1:**
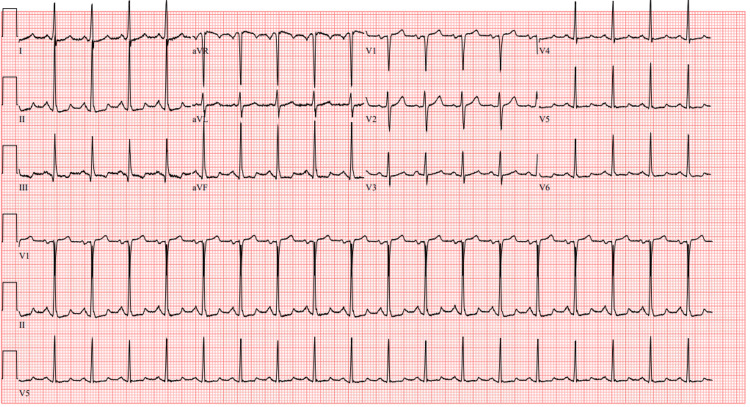
ECG ECG showing sinus tachycardia at a rate of 112 beats per minute and no acute ischemic changes.

Approximately four months later, she presented back to our ED with complaints of dizziness of two weeks duration, which was associated with palpitations and a feeling of presyncope upon standing. The patient reported trying meclizine, which did not provide relief. She also stated that she had occasionally noticed her HR at home increase to 150 beats/min. On presentation, her HR was 101 beats/min. The physical exam was completely unremarkable. At this time, laboratory investigation was unremarkable. ECG (Figure 1) noted sinus tachycardia of 112 beats/min and no concerning acute ischemic changes. The patient was advised to follow up with her PCP and was once again discharged home from the ED.

The following day, she returned to the ED again with complaints of resting chest pain and epigastric pain. The chest pain was characterized as radiating to the left arm and jaw, lasted almost an hour, and then self-resolved. Per the emergency medical service (EMS) report, on arrival at her home she was diaphoretic, nauseous, pale, and tachycardic with a HR of 150 beats/min and a point of care (POC) glucose level of 519 mg/dL; however, a 12 lead ECG taken at her home was negative for ST-elevation myocardial infarction (STEMI). In the ED, the physical exam was significant for a diaphoretic-appearing female with epigastric tenderness. Her vitals were notable for an HR of 135 beats/min, with systolic blood pressure (SBP) ranging in the 70s mmHg and diastolic blood pressure (DBP) ranging in the 50s mmHg, which was unresponsive to a two-liter bolus administration of normal saline. Lab investigations at this time revealed the following significant values (Table 2).

**Table 2:** Notable laboratory values from presentation Laboratory investigations show leukocytosis, lactic metabolic acidosis with a high anion gap, and significantly elevated troponin levels and lipase levels.

Parameters	Patient Values	Reference Range
White blood cell count	21.9 K/mcL	4.5-11 K/mcL
Platelet count	553 K/mcL	150-400 K/mcL
CO2	17 mmol/L	21-31 mmol/L
Glucose	406 mg/dL	70-105 mg/dL
Anion gap	16	5-15
Blood urea nitrogen	36 mg/dL	8-21 mg/dL
Creatinine	1.63 mg/dL	0.5-1.2 mg/dL
Aminotransferase	56 U/L	9-35 U/L
Magnesium	1.5 mg/dL	1.8-2.5 mg/dL
Lipase	373 U/L	22-51 U/L
Troponin	557 ng/L	0-14 ng/L
Lactate	2.5 mmol/L.	0.5-2 mmol/L
Beta-hydroxybutyrate	0.2 mmol/L	0.02-0.27 mmol/L
Blood cultures	No growth	No growth

**Figure 2:**
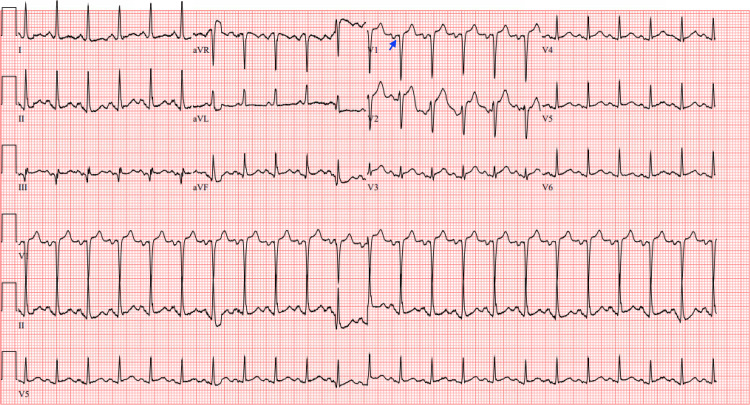
ECG ECG showing sinus tachycardia at a heart rate of 134 beats per minute and a Q wave in lead V1 (blue arrow).

The first ECG taken during this presentation (Figure 2) noted sinus tachycardia at a HR of 134 beats/min, no axis deviation, and a Q wave in lead V1.

**Figure 3:**
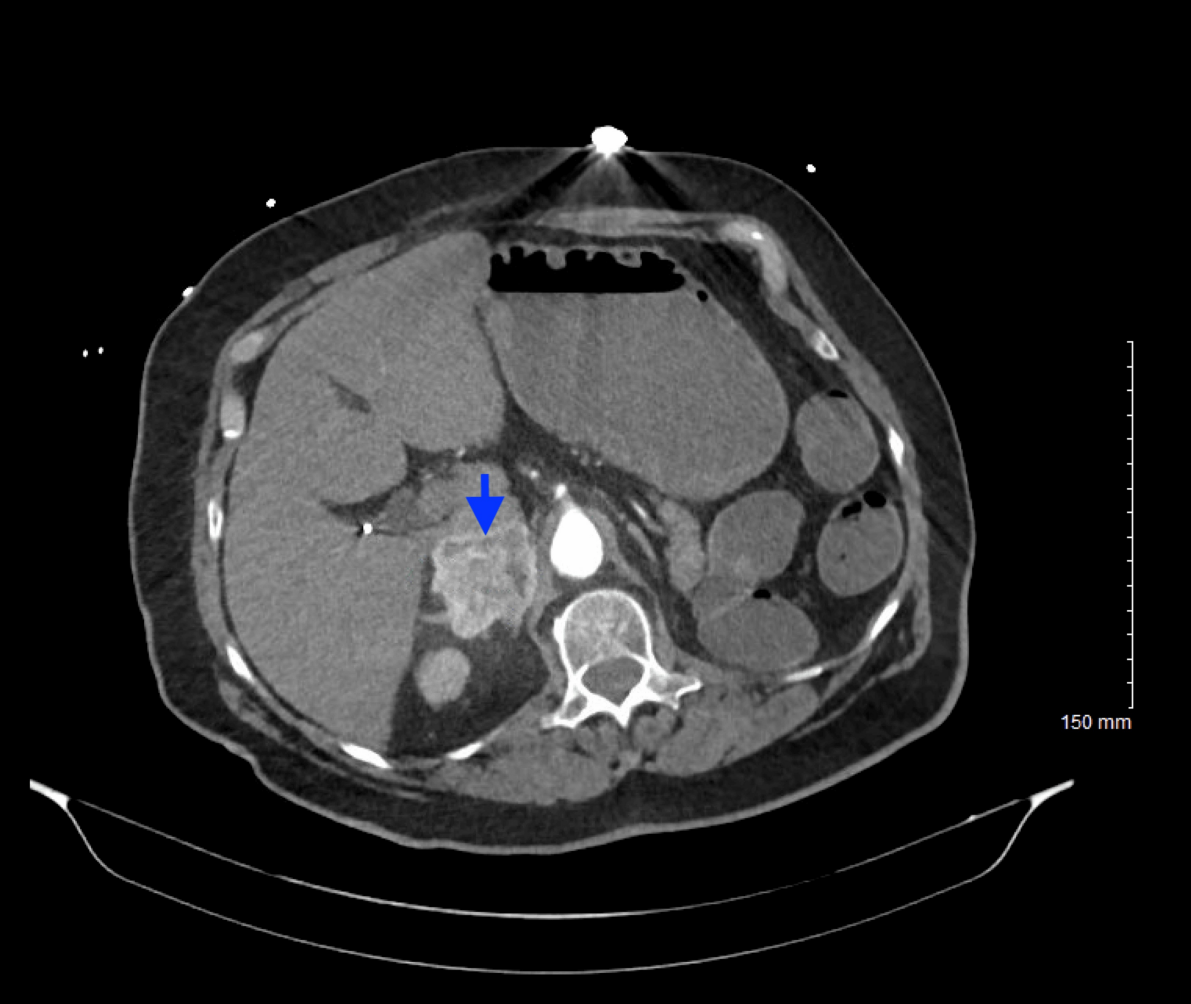
CT abdomen/pelvis with IV contrast (axial view) CT shows an arterial enhancing mass lesion (blue arrow) in the right retroperitoneal space measuring 4.2 x 4.3 x 5.5 cm.

**Figure 4:**
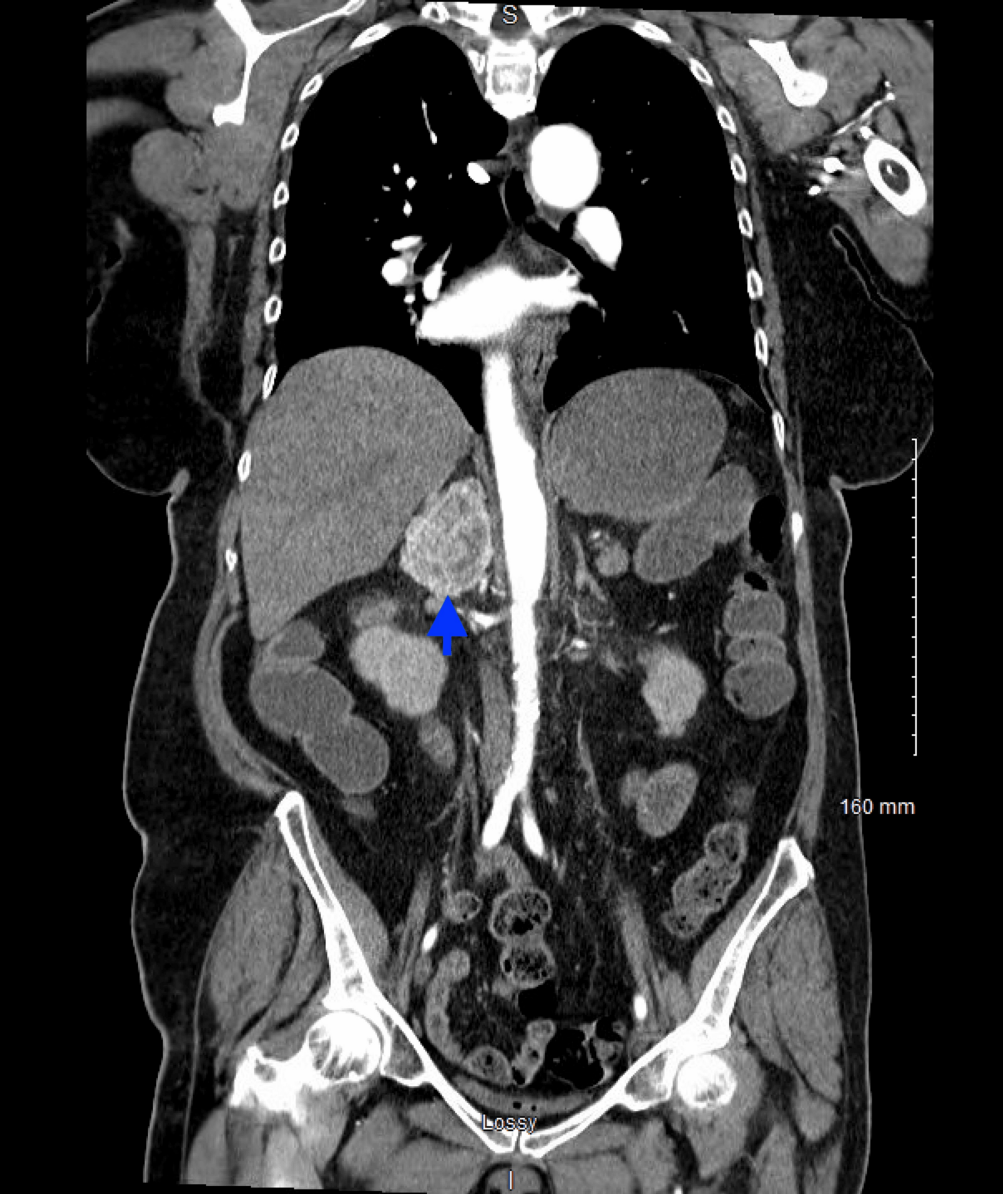
CT abdomen/pelvis with IV contrast (coronal view) CT shows an arterial enhancing mass lesion (blue arrow) in the right retroperitoneal space measuring 4.2 x 4.3 x 5.5 cm.

The CT angiogram of the chest/abdomen/pelvis was obtained due to suspicion of aortic dissection and was significant for an avidly arterial enhancing mass lesion seen in the right retroperitoneal space measuring 4.2 x 4.3 x 5.5 cm concerning for an adrenal pheochromocytoma and moderate coronary artery calcification (Figures 3, 4).

**Figure 5:**
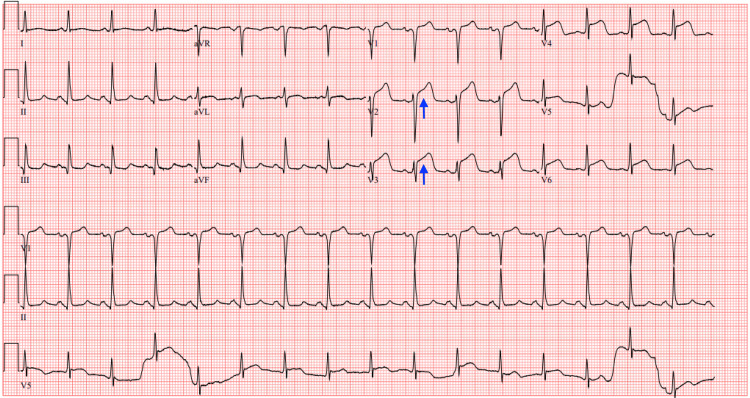
ECG (STEMI) ECG showing ST segment elevations in leads V2 and V3 (blue arrows). STEMI: ST elevation myocardial infarction

On repeat ECG, there were significant ST elevations evident in leads V2-V3 (Figure 5) for which both the intensive care team and the cardiology team were consulted, given the presence of hypotensive shock. She was urgently taken for coronary angiography which revealed 50-70% occlusion of the mid-segment of the left anterior descending (LAD) artery, a normal left ventricular ejection fraction (LVEF) of 60%, and an elevated left ventricular end-diastolic pressure (LEVDP) of 45 mm/Hg. No percutaneous coronary intervention (PCI) was done at this time; instead, the cardiologist recommended medical management with elective PCI to occur at a later date due to concerns about dual antiplatelet therapy (DAPT) after PCI causing hemorrhagic transformation of pancreatitis. She was admitted to the intensive care unit (ICU) for hemodynamic monitoring. Heparin and clopidogrel were considered to be of high risk to start at the time considering the presence of acute pancreatitis, lest they contribute to hemorrhagic transformation, so the patient only started receiving aspirin 81mg orally daily in combination with high-intensity statin therapy.

**Figure 6:**
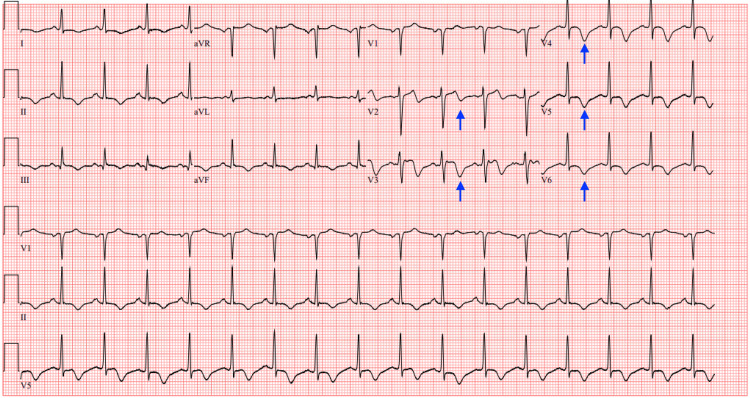
ECG ECG showing T wave inversions in leads V2-V6 (blue arrows).

The following day, however, after further discussion between the ICU team and the cardiology team, intravenous (IV) heparin drip was started peri-procedurally and the patient was noted to be requiring continuous norepinephrine infusion for cardiogenic shock. Overnight she started to complain of chest discomfort once again. ECG noted T wave inversions in leads V2-V6 (Figure 6), and an episode of non-sustained ventricular tachycardia was noted on telemetry. High-sensitivity troponin drawn that morning was 1833 ng/dL with a subsequent draw four hours later of 1692 ng/dL.

**Figure 7:**
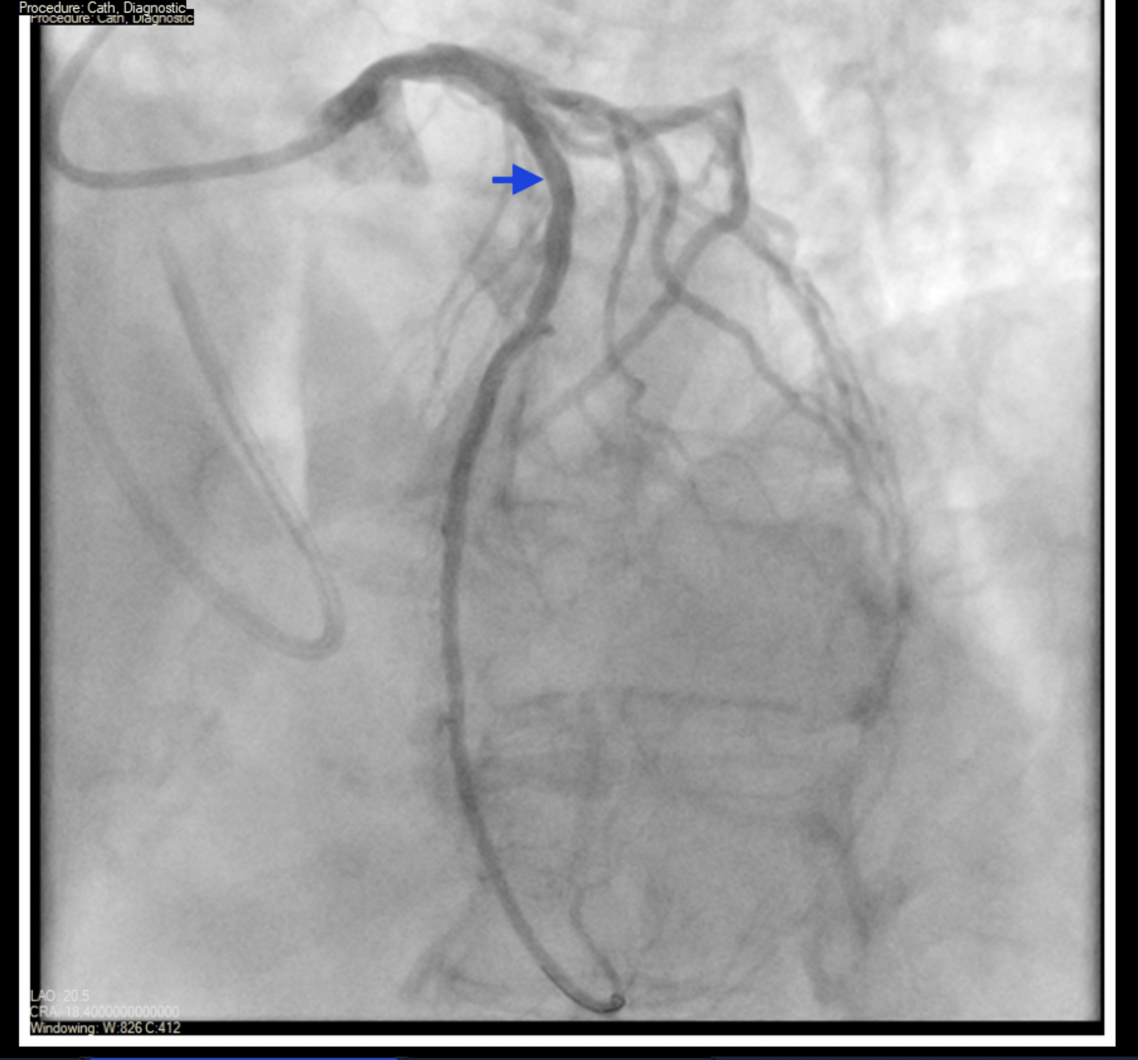
Coronary angiogram pre-PCI LAD (blue arrow) with 50-70% proximal and mid lesion stenosis with TIMI 3 flow and a minimal lumen area (MLA) of 5.4 mm^2^. PCI: Percutaneous coronary intervention

**Figure 8:**
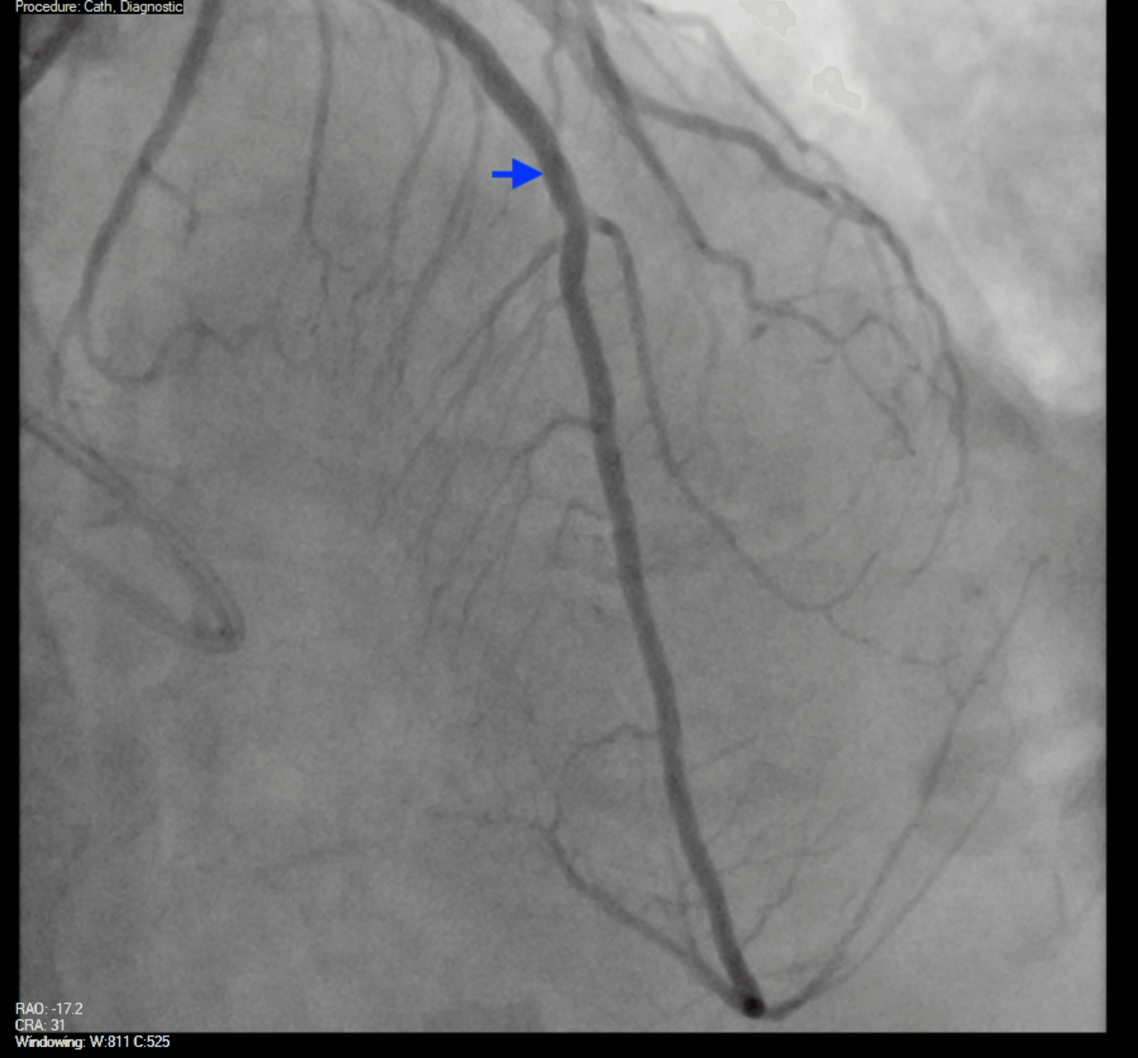
Coronary angiogram post-PCI LAD (blue arrow) with 0% residual stenosis, minimal lumen area (MLA) of 9.9 mm^2^, and TIMI 3 flow after stenting. PCI: Percutaneous coronary intervention

Due to her requiring ongoing IV vasopressor support, refractory angina, and findings consistent with non-ST elevation myocardial infarction (NSTEMI) on the ECG, she was taken back to the catheterization laboratory for coronary angiography and PCI, where a drug-eluting stent (DES) was successfully placed in the LAD ostia (Figures 7, 8). Ostial LAD had 50-70% stenosis with thrombolysis in myocardial infarction (TIMI) 3 flow and a minimal lumen area (MLA) of 5.4 mm^2 ^before stenting, which improved to 9.9 mm^2^ with 0% residual stenosis and TIMI 3 flow after stenting.

Afterwards, clopidogrel 75 g orally daily was started, and the patient was stable for transfer to medical floor. That evening, she experienced an acute episode of hypertension with BP 202/118 mm/Hg for which labetalol 10 mg IV was administered with BP returning to normal levels afterwards. Serum catecholamine levels were assessed and their values one week later are shown in Table 3.

**Table 3:** Serum catecholamine levels Serum catecholamine levels including metanephrine and normetanephrine were significantly high, thus consistent with the diagnosis of pheochromocytoma.

Parameters	Patient values	Reference range
Free metanephrine	60 pg/mL	<= 57 pg/mL
Free normetanephrine	4360 pg/mL	<148 pg/mL
Total free metanephrine + normetanephrine	4420 pg/mL	<205 pg/mL

**Figure 9:**
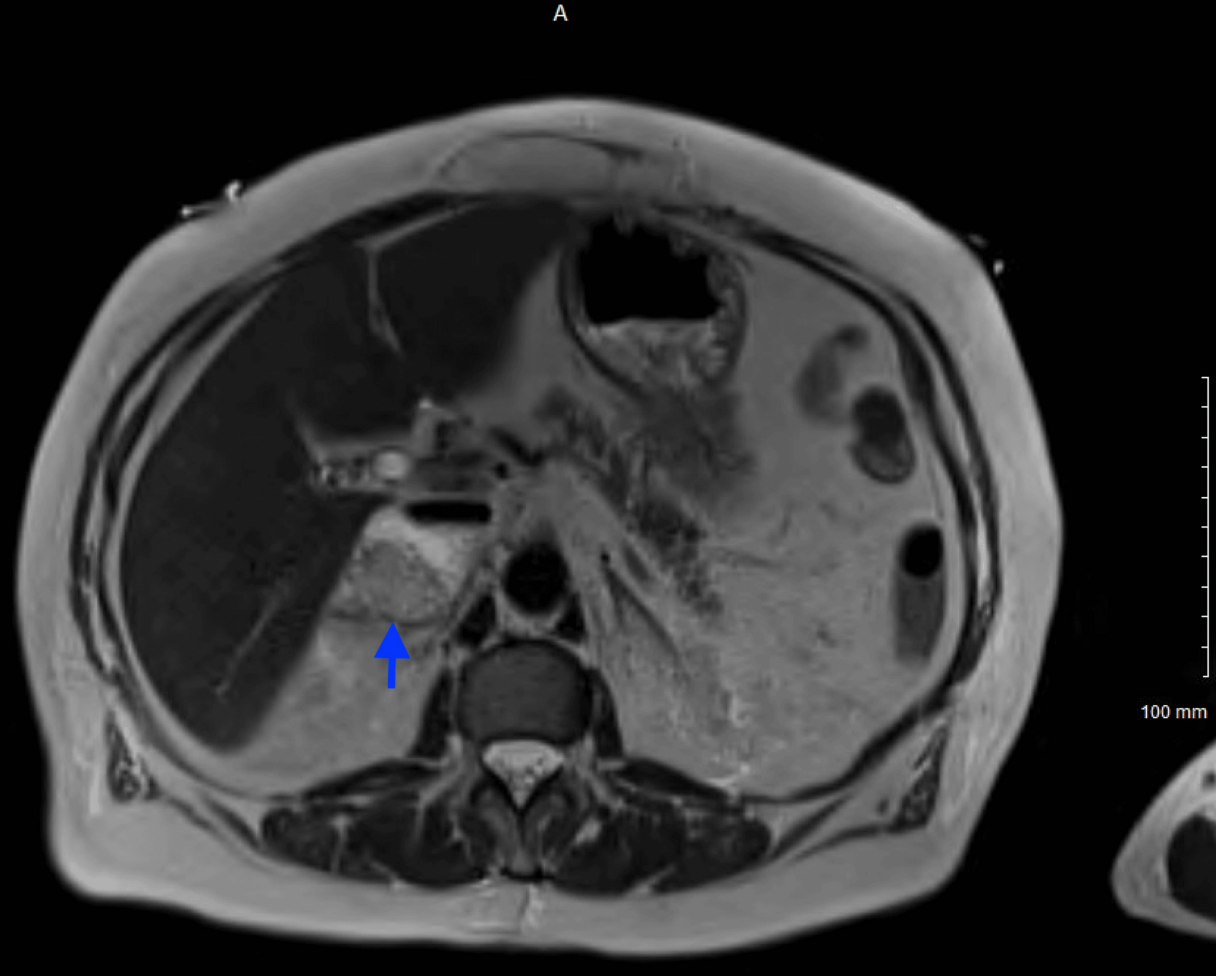
MRI abdomen without contrast T2 HASTE TRA-P2 series (axial view) MRI abdomen showing a lobulated, 5.8 x 4.6 x 3.8 cm heterogeneous mass (blue arrow) of the right adrenal gland.

**Figure 10:**
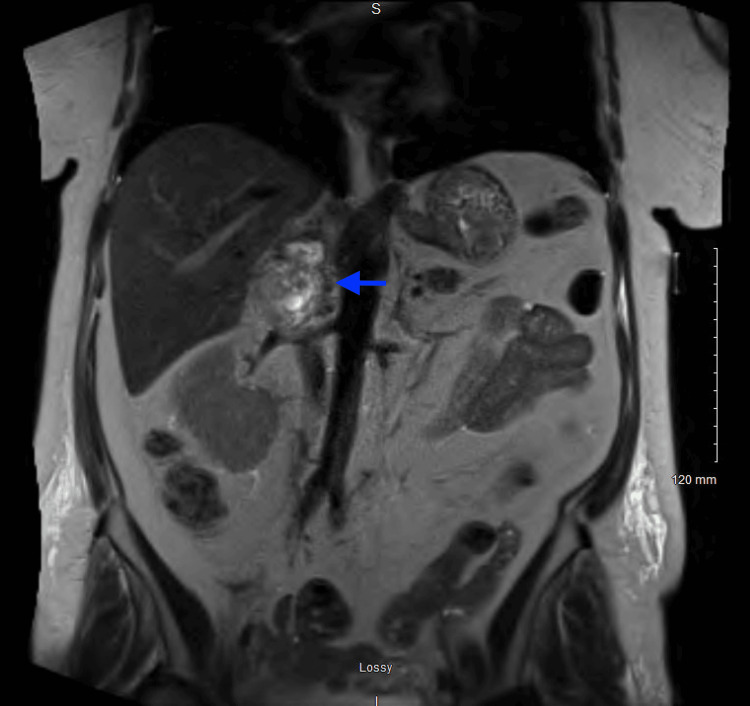
MRI abdomen without contrast - T2 HASTE series (coronal view) MRI abdomen showing a lobulated, 5.8 x 4.6 x 3.8 cm heterogeneous mass (blue arrow) of the right adrenal gland.

Magnetic resonance imaging (MRI) of the abdomen without contrast revealed a lobulated 5.8 x 4.6 x 3.8 cm heterogenous mass of the right adrenal gland; however, at that moment the etiology remained indeterminate (Figures 9, 10).

On day 4 of current hospital admission, once again she developed hypertension, diaphoresis, tachycardia, and chest discomfort for which the rapid response team was called. Chest discomfort and hypertension resolved after receiving three doses of sublingual nitroglycerin 0.3mg. Another ECG was taken during this episode that did not reveal any new acute ischemic changes. The cardiology team was contacted and recommended starting a nitroglycerin drip and transferring the patient back to the ICU. She was transferred to the ICU; however, the nitroglycerin infusion was never done as the chest pain did not re-occur and she was subsequently transferred to the medical floor on day 6 of admission. Due to labile BP throughout admission, prazosin was started for blood pressure control although it is unclear why other alpha-blocking agents such as phenoxybenzamine or phentolamine were not utilized. The endocrine team was consulted and recommended to continue with alpha-blocking agent prazosin, in addition to metoprolol for additional beta blockade with the goal of medical optimization in the setting of recent PCI and follow up in the outpatient setting by an endocrine surgeon. General surgery was also contacted during admission who recommended elective robotic right adrenalectomy once the patient recovers from the acute coronary event after obtaining cardiac risk assessment. Upon further discussion with cardiology, it was decided to continue with DAPT for at least 30 days with suspension of DAPT for seven days perioperatively. The patient was discharged home on day 14 of admission.

Twenty-six days later, the patient returned to the ED with complaints of substernal chest pain, radiating to the back, associated with diaphoresis, dizziness, palpitations, and shortness of breath. The patient’s husband stated that the patient’s BP on the onset of chest pain had systolic readings ranging between 180 and 200 mm/Hg and that she had taken a dose of prazosin 2mg orally without relief. On presentation, her HR was 117 beats/min, and she was noted to require six liters of supplemental oxygen per nasal cannula to maintain adequate oxygenation. Physical exam was significant for abdominal tenderness. An ECG did not reveal any new acute ischemic changes. Lab investigations were significant for the following values (Table 4).

**Table 4:** Significant lab values Lab investigations showing significant leukocytosis, and metabolic acidosis with a high anion gap. Kidney function is worsening based on creatinine levels, and troponin levels are elevated as well.

Parameters	Patient Values	Reference Range
White blood cell count	29 K/mcL	4.5-11 K/mcL
Hemoglobin	10.9 g/dL	12-16 g/dL
Platelet count	420 K/mcL	150-400 K/mcL
CO2	16 mmol/L	21-31 mmol/L
Anion gap	>20	5-15
Blood urea nitrogen	39 mg/dL	8-21 mg/dL
Creatinine	1.87 mg/dL	0.5-1.2 mg/dL
Lipase	432 U/L	22-51 U/L
Troponin	382 ng/L	0-14 ng/L
Brain natriuretic peptide	342 pg/mL	0-100 pg/mL
Lactate	2.1 mmol/L	0.5-2 mmol/L

CT angiogram (CTA) of the chest was obtained, which did not show any filling defects indicative of pulmonary embolism, neither any aortic pathology, and a subsequent CT of the abdomen and pelvis without contrast was obtained to evaluate for pancreatic pathology; however, it did not show any radiological signs of pancreatitis. The cardiology team was consulted again and did not recommend any invasive procedures. Instead, they believed it was best to continue with medical management including previously prescribed DAPT in addition to metoprolol and prazosin. The endocrinology team was reached out to once again and recommended outpatient follow up with an endocrine surgeon and to continue current medical management with Prazosin. The patient was successfully discharged home afterwards.

**Figure 11:**
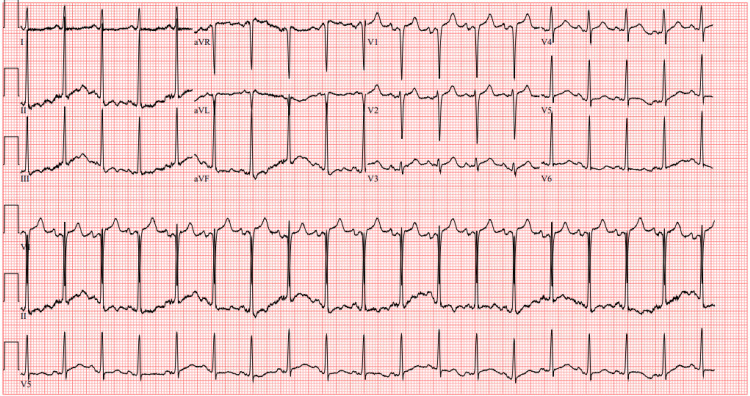
ECG ECG showing sinus tachycardia at a rate of 111 beats per minute, without any new acute ischemic changes.

Two weeks after that, she presented to the ED again with a similar picture and was noted to have significant wide anion gap metabolic acidosis where labs showed elevated lactate at 2.8 mmol/L, CO2 of 7 mmol/L, and AG > 20. ECG did not show ischemic ST changes (Figure 11). However, it did demonstrate sinus tachycardia. This required admission to the ICU and initiation of sodium bicarbonate infusion. During her ICU stay, she was noted to have multiple hypertensive symptomatic episodes which were managed with IV labetalol 5mg acutely and prazosin 1mg PO three times daily. However, the patient was not able to tolerate prazosin due to significant fluctuation in BP, so phenoxybenzamine 10 mg PO BID was started instead. Of note, after initiation of phenoxybenzamine, the patient stated her symptoms had significantly improved.

The endocrinology team recommended the patient be transferred to a tertiary care center for surgical adrenalectomy. On the fourth day of admission, she was transferred to a tertiary care center. On arrival, the endocrine surgery team evaluated the patient and were planning on moving forward with the surgery as planned; however, the cardiology team determined that the risk of stopping DAPT this early was higher than the benefit she would obtain from the surgery as it had seemed that significant symptomatic control was achieved with phenoxybenzamine. They recommended completing treatment with DAPT for at least 3-6 months before undergoing surgery. She was subsequently discharged from the hospital.

**Figure 12:**
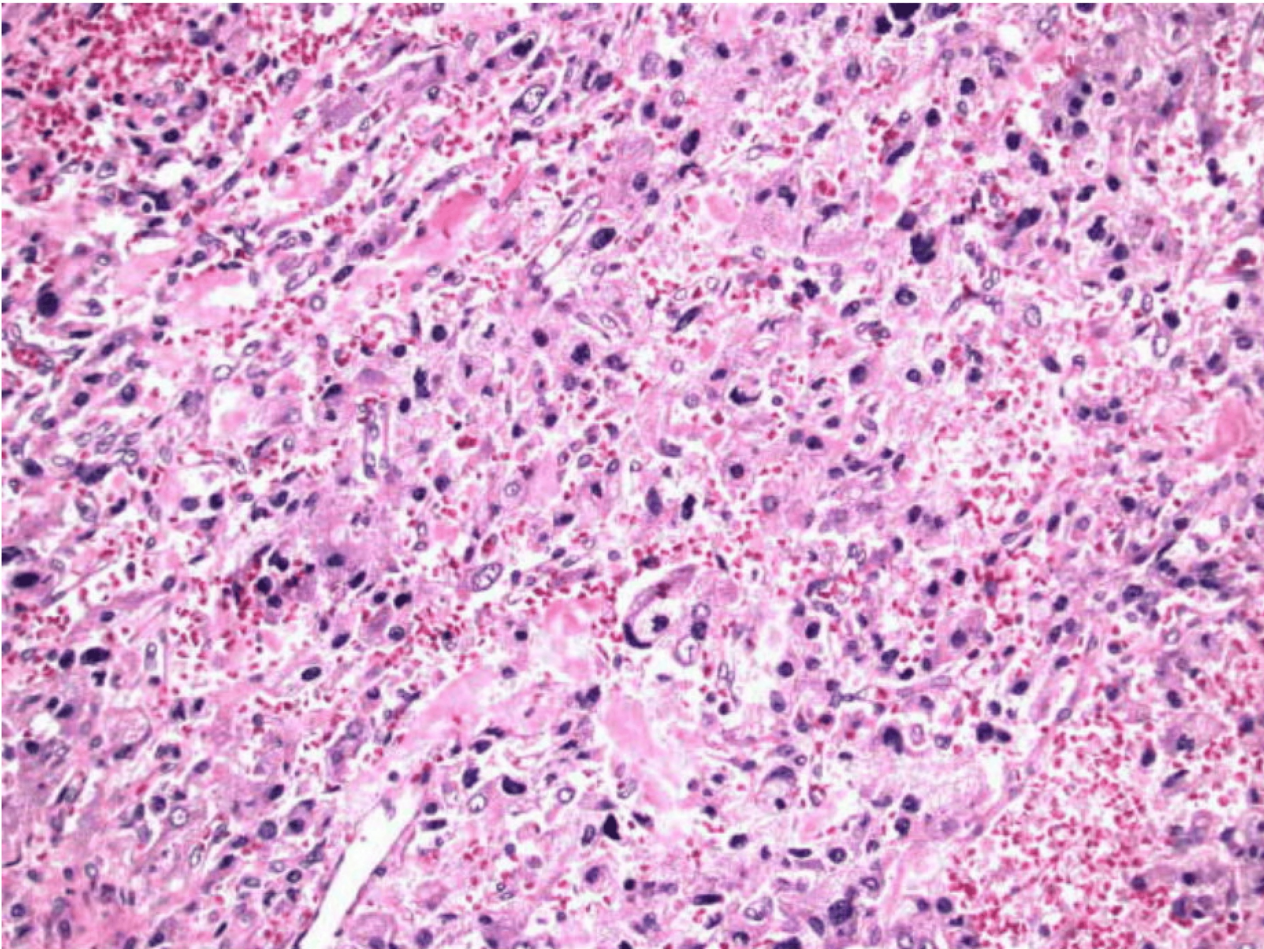
Histopathological examination of the surgical pathology sample consistent with the diagnosis of pheochromocytoma Histopathology sample showing a highly cellular, friable tumor composed of nests and sheets of polygonal tumor cells separated by a vascular stroma. The tumor cells appear to have abundant eosinophilic cytoplasm with round/oval nuclei. Mild to moderate nuclear pleomorphism is noted without atypical mitotic figures.

During this period, her symptoms were controlled, and she did not have any hypertensive episodes requiring admission to the hospital. Approximately six months later, she underwent robotic-assisted laparoscopic right adrenalectomy, which was complex given abutment of the inferior vena cava (IVC) and tedious dissection; however, the surgery was largely successful and kept minimally invasive. She was admitted to the surgical ICU given the need for vasopressor initiation for blood pressure maintenance post-operatively but rapidly improved and was transferred to the medical floor on postoperative day 2. She recovered well and was discharged home the following day. Findings from surgical pathology and immunohistochemical stains were consistent with pheochromocytoma (Figure 12, Table 5).

**Table 5:** Immunohistochemistry report of the surgical pathology sample Immunohistochemistry report of the surgical pathology sample showing findings consistent with diagnosis of pheochromocytoma.

Description	Score
Chromogranin	Positive
Synaptophysin	Positive
S100	Positive staining of sustentacular cells
GATA 3	Weakly positive
PAN CK	Negative
Ki-67	<1%, positive

Post-operatively, she had no subsequent complications and no admissions for similar symptoms. Hypertension medications have been discontinued as her ambulatory blood pressure measurements have returned to normal levels. She continues to follow up with her endocrinologist in the office setting.

## Discussion

Multiple case reports have been published reporting pheochromocytoma presenting as ACS [7-13]. These reported cases should urge physicians to maintain a broad differential diagnosis when encountering cases of ACS. While pheochromocytoma might not be a very frequent culprit when considering causes of ACS, it is certainly imperative that it be considered in cases where recurrent similar presentations have previously occurred, especially in a young patient who has no known cardiovascular risk factors, and when other features such as episodic headaches, palpitations, sweating and labile blood pressure are present.

The hypothesized mechanism behind presentation as ACS is due to sudden episodic release of catecholamines, which in theory might lead to significant blood flow limitation secondary to coronary artery vasospasm [8]. Another hypothesized mechanism would be catecholamine surge promoting hypercoagulability and thrombosis, which might prove to be detrimental in patients with already existing obstructive CAD in addition to catecholamine-induced myocardial stunning otherwise known as stress cardiomyopathy or Takotsubo syndrome [9]. This will result in myocardial ischemia and subsequent myocardial damage as evidenced by ischemic patterns on electrocardiogram and elevations in serum troponin levels. While there may not be an identifiable culprit vessel in some cases, there is always the possibility of overlap between pheochromocytoma and CAD, where obstructive lesions may be unmasked upon performing coronary angiography, especially in patients with other cardiac risk factors, including primary hypertension, diabetes mellitus, hyperlipidemia, obesity, and others.

Management of ACS due to malignant pheochromocytoma in the acute care setting may prove to be very challenging, considering administration of beta-blockade without pre-medicating with alpha-blocking agents (phentolamine or phenoxybenzamine) could hypothetically result in an unchallenged alpha adrenergic agonistic effect, thus possibly contributing to further damage. When using beta-blockers, it is preferred to use non-selective agents such as labetalol over selective agents like metoprolol. The use of contrast media during coronary angiography might also prove to be risky due to the possibility of precipitating hypertensive crisis or heart failure secondary to further catecholamine release; thus, the use of other modalities of cardiac imaging where non-ionic contrast or even no contrast is utilized might be considered [14]. The risk further extends to possible tumor hemorrhage secondary to use of anticoagulation peri-procedurally [12]. Last but not least, the choice of anesthesia in cases where adrenalectomy is planned is also important as the use of agents that increase the sympathetic tone such as ketamine, desflurane and pancuronium could result in further activation of the sympathetic response [13]. In similar cases, use of anesthetic agents that reduce sympathetic tone such as propofol might be more appropriate [15-17].

All of these challenges in acute management only complicate clinical decision making and might result in uncertainties or delays which could lead to further morbidity and mortality. While no clear clinical guidelines exist in these cases, the responsibility falls upon the clinician to consider all these risks and initiate an interdisciplinary team discussion regarding management on a case-by-case basis. Larger scale studies might be needed to provide any kind of direction or guideline toward management in these cases.

## Conclusions

Pheochromocytomas are rare endocrine tumors that can be very challenging to diagnose, as classic presentations are only present in a fraction of the cases. The fact that presentations of pheochromocytomas can mimic other non-cardiac conditions such as panic attacks, thyrotoxicosis, illicit substance ingestion only serves to complicate clinical assessment and decision making. These tumors have been reported to present as ACS with or without pre-existing CAD. Diagnosis requires a high degree of suspicion, and management in the setting of ACS can prove to be very difficult, especially if there is no known history of pheochromocytoma in the acutely presenting patient. Missing or delaying diagnosis or management in these cases might prove to be detrimental to the patient, as complications can occur at any time, thus urgent evaluation of such cases is worthwhile.

Clinical practice guidelines have been in use for a long time in the management of ACS; however, the presence of underlying pheochromocytoma can lead to unexpected complications that are best managed by an interdisciplinary medical approach involving specialists from fields including internal medicine, endocrinology, surgery and others like cardiology, critical care and anesthesiology when appropriate. Physicians must consider a broad differential diagnosis when encountering cases of ACS, particularly whenever encountering unusual components of the presentation such as young age, absence of cardiovascular risk factors, recurrent episodic presentations, history of paroxysmal tachyarrhythmias and labile blood pressure. We anticipate the need for more studies to provide recommendations for acute and chronic management of such cases. Observational cohort studies might be a good starting point to shed light on different outcome rates using different management techniques.
